# Urinary tract infection in cancer patients in a tertiary cancer setting in India: microbial spectrum and antibiotic susceptibility pattern

**DOI:** 10.1186/2047-2994-4-S1-P221

**Published:** 2015-06-16

**Authors:** P Parikh, V Bhat

**Affiliations:** 1 Tata Memorial Centre, Mumbai, India; 2 Tata Memorial Centre, Mumbai, India

## Introduction

In immunocompromised cancer patients, urinary tract infection (UTI) is one of the major causes of fever and morbidity. Screening for UTI is important as atypical presentation is not uncommon in such patients.

## Objectives

To determine the common organisms implicated in UTIs in cancer patients and to study their antimicrobial susceptibility patterns. This would help formulate empirical antibiotic policy in this group of patients.

## Methods

A retrospective analysis of cancer patients suspected to have UTI in the year 2014 was carried out. A total of 497 midstream urine samples collected from cancer patients suspected to have UTI, were sent to the microbiology lab for urine routine and culture examination. Samples were processed as per standard microbiological procedures. All isolates were identified up to species level and antimicrobial susceptibility tests performed as per CLSI guidelines.

## Results

Of the 497 samples processed, 100 were positive for bacterial growth. Overall, *E.coli* (40%) was the predominant isolate followed by *Klebsiella pneumoniae* (25%), *Pseudomonas aeruginosa* (11%), *Enterococcus spp* (11%) and *Proteus mirabilis* (5%). Susceptibility of Gram negative bacteria to colistin was highest (100%) followed by the carbapenems (72%). Resistance was found to be higher to the aminoglycosides (46%), cephalosporins (67%) and fluoroquinolones (90%)

## Conclusion

E. coli was the most common organism isolated in cancer patients with UTI. There is trend of increasing resistance to aminoglycosides, cephalosporins and fluoroquinolones among Gram negative bacilli.

## Disclosure of interest

None declared.

